# In memory of Allan William Cripps AO (1950–2022)

**DOI:** 10.1186/s41479-023-00107-7

**Published:** 2023-02-16

**Authors:** Maree Gleeson, Amanda Cox, Nicholas West

**Affiliations:** 1 University of Newcastle, Newcastle, Australia; 2 Menzies Health Institute Queensland and School of Pharmacy and Medical Sciences, Griffith University, Parklands, Australia

Professor Allan Cripps AO, an internationally recognised expert in the field of mucosal immunology and a leading public health administrator, passed away on 20 December 2022. Allan is celebrated as the founding editor of this Journal, Pneumonia, as a passionate and engaging scientist, an advocate for improving access to education for disadvantaged students and for the development of one of Australia’s largest University health and medical teaching and research programs.

Allan grew up on a farm in remote New South Wales and as a boy from the bush kept rural health and rural access to education close to his heart. His schooling was largely by correspondence and he was awarded an Australian Commonwealth Scholarship, without which he acknowledged would have meant he would have been unable to attend University. He would complete his PhD in immunology at the University of Sydney, focusing on defining aspects of the host-parasite relationship at mucosal surfaces and the mechanisms by which antibody is delivered to mucosal sites. These studies provided evidence to support the hypothesis of a common mucosal immune system and his discoveries formed the basis of our understanding of IgA secretion to surfaces of the body.

After completing a post-doctoral fellowship in Sweden, Allan returned to Australia in 1976 to lead diagnostic immunology services, initially at Flinders Medical Centre in Adelaide and then at the Hunter Area Pathology Service in Newcastle, during the period of establishment of the Medical Faculty at the University of Newcastle. He continued his research in mucosal immunology focusing on bacterial infections in children. From 1979 to 1985, he led the most comprehensive longitudinal study ever conducted on the development of mucosal immunity in infants and children. This research informed the future development of mucosal vaccines to prevent childhood respiratory infections and played a pivotal role in understanding the role of respiratory infections in sudden infant death syndrome. Allan’s research laid the foundation for the biotechnology essential to the investigation of mucosal immunity and these assays have been adopted worldwide for the purification of proteins, for vaccine development and immunoassays for human and animal studies.

Allan held the several positions at the University of Canberra between 1992 and 2003 including Dean of the Faculty of Applied Sciences and Pro-Vice Chancellor of Research and International. During this period, Allan’s expertise in mucosal immunology and interest in the gut microbiome saw him lead pioneering studies in respiratory health in elite athletes with the Australian Institute of Sport.

In 2003, Allan was appointed the inaugural Pro Vice Chancellor (Health) that established the Griffith University School of Medicine for the Gold Coast. His ability to bring individuals together from science, industry and medicine led to the Health Group becoming the largest academic group at the University and in setting up a rural health program. During his tenure as PVC, Allan led the Mucosal Immunology Research Group that continued his work on mucosal immunity, vaccine development and diagnostic technology. Allan published over 400 papers and mentored 27 doctoral candidates. On retiring, Allan was made Emeritus Professor at Griffith University.

Allan held multiple leadership positions in research and academia. He founded the Australian Society for Immunology’s Special Interest Group for Mucosal Immunology (MI-SIG) and served as its Chair for 15 years. To this day, the MI-SIG provides a forum for mucosal immunologists and colleagues with associated interests to meet via workshops, symposia and satellite meetings, to share and progress their research work and collaborations, and has supported new researchers in the field of mucosal immunology.

Allan’s intellectual drive and passion for mucosal immunology and health continued in retirement and he remained an active scientist. Allan was a fellow of the Australian Academy of Health and Medical Scientists, the Australian Society for Microbiology, the Australian Institute of Medical Scientists, the Institute of Biomedical Scientists (UK) and the Australasian College of Health and Service Management. Allan was the holder of many patents and had considerable commercial experience in medical biotechnology. He served as a director and Chair of many health, government and industry boards.

In 2015, Professor Cripps was awarded the Order of Australia for distinguished service to tertiary education as a senior administrator and to public health as a leading immunologist, academic and researcher in the area of mucosal immunology.

For those that worked with Allan, we celebrate a man of great warmth, kindness and compassion, insight of character and wit, and who was an inspiration to all around him. Many of us were privileged to share meals at his home while he opened the treasures of his wine cellar. He will be remembered with great respect and fondness.

Allan is survived by his wife Diana and their two children, Zachary and Sofie, without whose support and love his achievements would not have been possible, and three children from his first marriage, Nathan, Dane and Natasha.

